# On the Dynamical Regimes of Pattern-Accelerated Electroconvection

**DOI:** 10.1038/srep22505

**Published:** 2016-03-03

**Authors:** Scott M. Davidson, Matthias Wessling, Ali Mani

**Affiliations:** 1 Department of Mechanical Engineering, Stanford University, Stanford, CA 94305, USA; 2 AVT Chemical Process Engineering, RWTH Aachen University, Turmstraße 46, 52064 Aachen, Germany; 3 DWI – Leibniz-Institute for Interactive Materials, Forckenbeckstraße 50, 52074 Aachen, Germany.

## Abstract

Recent research has established that electroconvection can enhance ion transport at polarized surfaces such as membranes and electrodes where it would otherwise be limited by diffusion. The onset of such overlimiting transport can be influenced by the surface topology of the ion selective membranes as well as inhomogeneities in their electrochemical properties. However, there is little knowledge regarding the mechanisms through which these surface variations promote transport. We use high-resolution direct numerical simulations to develop a comprehensive analysis of electroconvective flows generated by geometric patterns of impermeable stripes and investigate their potential to regularize electrokinetic instabilities. Counterintuitively, we find that reducing the permeable area of an ion exchange membrane, with appropriate patterning, increases the overall ion transport rate by up to 80%. In addition, we present analysis of nonpatterned membranes, and find a novel regime of electroconvection where a multivalued current is possible due to the coexistence of multiple convective states.

Chaotic electroconvection is a recently discovered microscale hydrodynamic phenomenon that exhibits many features commonly associated with turbulence despite having zero Reynolds number. Specifically, this phenomenon involves highly unsteady vortices with broadband spectra over a wide range of scales. However, since all length scales involved are small, chaotic electroconvection is not influenced by inertia. Instead, these flow features are generated by complex, nonlinear interactions between charge transport, electric fields, and fluid motion. In such settings, the applied electric forcing plays a role similar to the Reynolds number, governing transitions through stable, laminar unstable, and finally to fully chaotic regimes. Unlike traditional turbulence which has been studied extensively for many decades, electroconvective “turbulence” has only come to light recently with the first canonical numerical simulations published in the last five years^1^ and extensive quantitative experiments published this year^2^.

Electroconvection is crucial to overcoming transport barriers due to diffusive limitations governing vital desalination and energy conversion processes by mixing the fluid, playing a similar role to that of turbulence in classical heat and scalar transport problems. The diffusive transport of ions to charge-selective surfaces is rate-limiting in many electrochemical systems of both industrial and academic interest. In the absence of electroconvection, flow batteries, electrodialysis, electrolysis, and electrodeposition all experience saturation of transport to a limiting current as the concentration at a selective surface approaches zero, due to the known concentration polarization phenomenon^3^. This limiting behavior is often quantified by measurement of current versus voltage (IV) curves which exhibit characteristic plateau regions where current does not increase with voltage. However, with further increases in the driving voltage above a critical threshold, overlimiting current (OLC) is observed as the current again rises. The mechanisms governing OLC have been investigated by many authors, and recently a consensus has been established that transport due to electroconvection which arises as a result of electrokinetic instability (EKI) of the fluid very near the ion-selective surface is the dominant source of OLC in these systems^4^.

EKI is not the only method of generating electroconvection. Variations in surface properties such as ion-permeability^5^, surface charge^6^, geometry^7^,^8^, and reactivity^9^ also induce electroconvection. In these cases the surface heterogeneity leads to components of the electric field tangent to the surface, which act on the charged layer near the surface to induce flows. Standard heterogeneous membranes display local limiting and overlimiting currents several times the average of homogeneous membranes, but with lower surface-averaged current densities^10^. However, recently, nanometer thick polyectrolyte layers deposited in micropatterns on ion exchange membranes were found to significantly shorten the limiting plateau length in the IV curve and increase OLC^11^. Thus, understanding the interactions between these two modes of electroconvection provides a promising avenue for designing surfaces that enhance OLC.

Rubinstein and Zaltzman^7^,^12^,^13^ provided the foundation for the current understanding of OLC by performing the linear stability analysis of coupled hydrodynamic and ion-transport equations subject to an applied electric field. This instability was observed directly at a membrane surface by Rubinstein *et al*.^14^. A number of computational works performing direct numerical simulation (DNS) of the coupled Poisson-Nernst-Planck and Navier-Stokes equations have investigated the nonlinear transition with increasing applied voltage from steady vortices^15^,^16^ to fully chaotic flow^1^,^17^,^18^,^19^. Despite this work, chaotic electroconvection, due to its complex and multiscale nature, remains poorly understood. Experimentally, its turbulent footprint is observed as noise in voltage signals. Theoretically, deeper knowledge emerges only slowly as work is done in various contexts using direct numerical simulation to investigate the effects of wavy surfaces^20^ and cross-flow^21^,^22^ as well as the discovery of the potential for EKI in induced-charge electroosmosis^23^.

Very recently, de Valença *et al*. reported comprehensive experimental measurements of EKI at a homogeneous membrane^2^. Using particle image velocimetry, they quantified vortex size growth rates and vortex speeds. Additionally, they showed images of seeding particle motion at various times in the vortex development. We translate, using virtual colloidal particles, simulation results reported in this paper into data resembling the experimental images as shown in Fig. 1. Strikingly similar tracer-free void regions are present in both simulated and experimental images. Despite the absence of tracers, there is significant flow in these regions. The voids, which were not explained experimentally, arise from the inability of particles to accurately act as tracers very near the surface. In our simulations, very large electric fields near the membrane exclude even the almost uncharged (*ζ* = −1 mV) virtual particles. This unprecedented ability for direct comparison of experimental vortex visualization and simulations of electroconvection will be important to validate the results of simulations and build confidence in CFD codes used for prediction of electroconvection.

Considering broader applications involving turbulence, decades of research has allowed for development of surface modifications both active and passive to control turbulence and the instabilities that lead to it. Turbulent drag reduction on the order of 10% has been achieved via surface patterning with riblets^24^. Superhydrophobic surfaces utilizing trapped air pockets have shown drag reductions approaching 50%^25^. In Rayleigh-Benard convection, arrays of heating elements have been used to actively control the growth of convective instabilities^26^.

Inspired by the above examples from classical turbulent flows, in this study we investigate control of electroconvective “turbulence” via design of surfaces with patterned ion-permeability. We demonstrate the ability of properly sized patterns to regularize electroconvection and significantly enhance overlimiting transport. Specifically, we investigate small and large pattern wavelengths and demonstrate that wavelengths on the order of the diffusion boundary layer can lead to an enhancement in ion transport rate approaching 80%. This magnitude of increase is remarkable when compared to what has been achieved with passive control of classical turbulent flows. Additionally, our investigations reveal for the first time that in the case of homogeneous unpatterned membranes, at voltages slightly above the onset of instability, two modes of transport with distinct flow features can coexist. Due to this coexistence, a multivalued I-V curve results for a portion of the parameter space.

## Results

### Model Problems

We compare two model problems each consisting of a symmetric binary aqueous electrolyte solution between two cation-selective surfaces separated by a distance 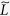 across which a potential difference 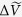 is applied as shown in Fig. 2. They differ in the boundary conditions at the depleted (lower) surface. The first is a homogeneous cation exchange membrane while the second is a patterned surface with alternating ion-permeable 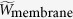 and impermeable 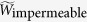 regions of period 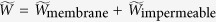. In both systems, the enriched (upper) surface is modeled as a homogeneous cation exchange membrane. Pattern asymmetry is characterized by the fraction of each pattern period consisting of membrane, 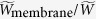.

With the unmodified membrane surface, the only mechanism for electroconvection is EKI, the so-called Rubinstein type electroconvection which occurs due to instability above a critical threshold voltage. The patterned surface, because of its inhomogeneity, induces electroconvection at any applied voltage^5^. When a potential is applied across the cell, screening electric double layers (EDLs) form on the impermeable regions causing electric field lines to curve around these zones and be focused in the membrane regions as seen in Fig. 2(d). The tangential component of the electric field then acts on the EDL itself inducing electroosmotic flow away from the center of the impermeable element and, because of continuity, generates a pair of counter-rotating vortices on each impermeable region. Near the limiting current, the EDL becomes non-equilibrium and the electroosmotic flow transitions from first to second kind, the so-called Dukhin type^3^,^27^. Above a threshold voltage, the non-equilibrium EDL becomes unstable and there is interaction between the pattern-induced and instability-induced modes.

### Governing Equations

The system, in a dilute limit, is governed by the coupled Poisson-Nernst-Planck equations for the electric potential and ion transport respectively and Navier-Stokes equations for the fluid momentum. Ignoring the nonlinear inertial term in the NS equations due to the very small Reynolds number of the system, these equations become


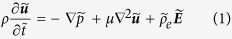



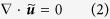



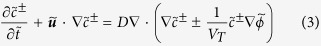



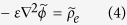


where the electrolyte has density *ρ*, velocity 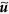, dynamic viscosity *μ*, and dielectric permitivity *ε*. 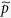 is the pressure, 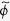 is the electric potential, 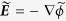 is the electric field, and 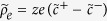 is the free charge density. The ionic species have number density 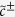 and diffusivity *D. V*_*T*_ is the thermal voltage defined as *V*_*T*_ = *k*_*B*_*T*/(*ze*) where *k*_*B*_, *T, z*, and e are Boltzmann’s constant, temperature, ionic valence number, and the elementary charge respectively. For a monovalent electrolyte at room temperature, 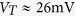, i.e. 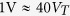.

These equations are nondimensionalized with length, time, velocity, pressure, potential, and concentration scaled by the cell height 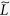, diffusion time 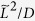, diffusion velocity 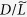, viscous pressure 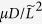, thermal voltage, and mean anion concentration 
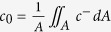
 which is constant in time for both systems. Dimensionless quantities correspond to dimensional quantities with the tilde removed. Nondimensionalizing gives four governing dimensionless parameters, the electrohydrodynamic coupling constant *κ*, dimensionless Debye length *ϵ*, Schmidt number Sc, and dimensionless applied voltage Δ*V* where


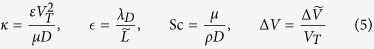


with *λ*_*D*_ being the Debye length defined as 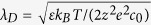 over which screening of equilibrium EDLs occurs. Two of these four governing parameters, *κ* and *Sc* are fixed by the choice of electrolyte. We use values of *κ* = 0.5 and Sc = 1000 corresponding to typical aqueous electrolytes. *ϵ* is typically small, ranging from 10^−7^ for a 0.1 M electrolyte in a 1 cm system to 10^−3^ for a 1 mM electrolyte in a 10 *μ*m system. In this study we consider *ϵ* = 10^−3^ which is sufficiently small to be in the regime of interest while allowing for a reasonable computational time. The applied voltages we consider are Δ*V* ~ 1 − 100*V*_*T*_.

Dimensionless boundary conditions at the membrane surfaces are given by


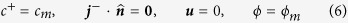


where *c*_*m*_ is a constant representative of the membrane surface charge, ***j***^−^ is the anion flux. Ion fluxes are given by 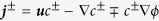, and *ϕ*_*m*_ is the membrane potential with *ϕ*_*m*_ = 0 at *y* = 0 and *ϕ*_*m*_ = Δ*V* at *y* = 1. The species transport boundary condition which ignores co-ion penetration into the membrane is asymptotically valid for 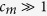
13. However, it has been found that the solution is nearly independent of *c*_*m*_ for even *O*(1) values thus we choose *c*_*m*_ = 2 1. For the patterned system, the boundary conditions on the surface are given by


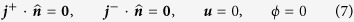


Due to our choice of *ϕ* BC, the impermeable regions represent an inert conducting material. In the tangential *x*-direction, ideally an infinite domain would be used. We approximate this numerically with a periodic boundary condition and use sufficiently large domain sizes to ensure convergence with aspect ratios varying from 4 to 64. Initial conditions used are uniform species concentrations with a zero-mean *O*(10^−6^) random perturbation added and quiescent flow.

### Unpatterned: Transition to Chaos

The unpatterned system exhibits qualitatively different behavior in several voltage regimes as it transitions to chaos with increasing applied voltage. This process was investigated using both DNS and weakly nonlinear analysis in^17^ and^18^. Here we describe a novel secondary plateau in the current vs voltage curve in Fig. 3(c) as well as the existence of a range of possible steady-state currents in an intermediate voltage regime indicated by the shaded region of Fig. 3(c). The flow structures observed during this transition are described below.

The system undergoes a subcritical bifurcation at a critical voltage, 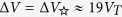
17 transitioning from the 1D solution to a 2D regime in a hysteretic manner^14^,^16^. Even small increases in Δ*V* relative to 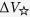 lead to a nonlinear vortex size selection process occurring until a periodic steady-state is reached consisting of vortex pairs at a period close to 2*L*. As vortices pair, they lose their initial sinusoidal character and instead spike-like charge density structures are seen generating regions of strong outflow as shown in Fig. 2(c). The same behavior occurs at Δ*V* = 25*V*_*T*_, as in Fig. 3(a).

At higher voltages 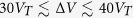, however, a new regime is observed where multiple steady-state vortex sizes coexist after simulations were run for many diffusion times 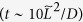. The boxed regions in Fig. 3(d) highlight an example scenario from a calculation over a domain with very large aspect ratio (32) in which small and large vortex pairs coexist. Coexistence of multiple wavelengths in discrete regions is also seen in other instabilities including Rayleigh-Bénard Convection^28^ and circular Couette flow^29^.

We found the fraction of the domain covered by each vortex size to not be unique and to be dependent on the initial perturbations. However, in all of these simulations, zones of the same vortex size were observed to have the tendency to stabilize (attract) each other. Therefore, in the long-time limit, a process similar to phase separation was observed in which bigger blobs of multiple vortices of the same size were found to be more common than isolated vortex pairs of a certain size surrounded by vortices of the other size. Interestingly, each vortex size generates a distinct local current density. The net mean current density is therefore not unique, and depends on the selected composition of vortices. Figure 3(c) shows mean current density given by 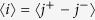, where the mean is surface-averaged and time-averaged over a long time after the initial transition. Here each symbol (circles in Fig. 3(c)) at a given voltage represents a single simulation with only the small random initial perturbation changed between simulations. The shaded region represents an approximate range of possible mean current densities, while the upper and lower bounds, indicated by the dashed lines, are extracted from conditional means over zones of large and small vortices respectively. Details of the calculation of these values as well as vortex size distribution statistics are provided in the supplementary information.

The large vortices drive much larger currents, by a factor of approximately 2, via two mechanisms. One is the interaction between the vortices and the background concentration gradient. Larger vortices more effectively transport enriched fluid from close to the upper membrane to the depleted lower surface. The second is the conservation of total anion concentration imposed by the no flux BCs at both surfaces. This causes the concentration of quasi-electroneutral salt just outside of the upper EDL to vary spatially, and it is higher where the larger vortices are present. Thus, the vortices are acting not simply on a fixed background concentration gradient as might be expected from the 1D solution. Instead they act on a spatially varying effective concentration gradient strongly affected by the vortices themselves.

Large simulation domain aspect ratios (up to 64) were required to quantitatively capture the coexistence of two-vortex-size solutions. Previous investigations^15^,^16^,^17^ have likely missed this regime due to their very small aspect ratios (≤2*π*). We note that, while at Δ*V* = 30*V*_*T*_ the system reaches a long-term steady state, at Δ*V* = 35*V*_*T*_ and 40*V*_*T*_ the long-term solution involves finite amplitude oscillations due to emergence of smaller vortices closer to the surface induced by the high-wavenumber instability of the non-equilibrium EDL^1^ as well as side-to-side bouncing of the smaller vortices. These oscillations modulate the two-vortex-size solution but do not change the aforementioned qualitative observations.

At Δ*V* = 45*V*_*T*_, after an initial transient, all of the persistent large-scale vortices are of a single wavelength (i.e. no coexistence of two vortex sizes) while small-scale instability persists on the membrane surface. For Δ*V* = 50*V*_*T*_ and above, the system enters a fully chaotic regime, shown for Δ*V* = 80*V*_*T*_ Fig. 3(b), where current scales linearly with the applied voltage analyzed by Druzgalski and coworkers^1^. In the supplementary information, we provide Supplementary Movies S1,S2,S3,S4 showing the evolution of concentration fields and pathlines for a range of voltages as well as surface-averaged current versus time plots for these scenarios in Supplementary Fig. S2.

### Effects of Pattern Size

We investigate the flow structures generated by symmetric surface patterns of varying periods and the interactions of the pattern-generated flows with EKI flows. Figure 4 shows salt *c* = (*c*^+^ + *c*^−^)/2 concentration fields comparing effects of patterns with different wavelengths in reference to unpatterned results obtained at Δ*V* = 20 (left column) and Δ*V* = 60 (right column).

For the lower applied potential, the unpatterned system is barely above its threshold potential for instability. Only for long times does the system reach the steady-state shown in Fig. 4(a) consisting of counter-rotating vortex pairs. The patterned systems, Fig. 4(c,e), in contrast are stable. The patterned surfaces induce tangential electric fields at any voltage and thus generate electroosmotic flows in a thresholdless manner (Fig. 2(d)). This modification of the base state induced by the pattern increases the threshold potential required for instability. The pattern-induced vortices are of the same period as the pattern itself, however, the vortex size is constrained by the channel height when the wavelength of the pattern becomes large (*W* > 2*L*). Accordingly, a pattern with a period that is twice the channel height Fig. 4(e) induces vortices of the same size as the channel. This leads to efficient transport of ions globally across the entire channel, from the enrichment side to the depletion side. Patterns significantly smaller than the channel height Fig. 4(c) however induce small vortices which merely mix the depleted fluid, and thus do not generate significant OLC in the steady regime. Furthermore, small patterns disrupt the formation of large vortices which would otherwise form on a homogeneous surface as a natural consequence of EKI Fig. 4(a).

At 60*V*_*T*_ all of the systems are in the chaotic regime, exhibiting aperiodic fluctuations. The small pattern Fig. 4(d) doesn’t influence the stronger EKI formed at the higher voltage, and thus the flow is very similar to the unpatterned surface shown in Fig. 4(b). The large pattern, in contrast, induces flows which interact strongly with the EKI regularizing the flow. Because of these advantageous characteristics of symmetric patterns with period about twice the channel height, a pattern period of 2 was chosen for all further simulations in this study. While some aspects of pattern optimization are demonstrated here, we leave the fine-tuning of voltage-dependent pattern optimization to a future publication.

### Current Amplification via Surface Patterning

Figure 5 contains a comparison between the current versus voltage curves of the system with a patterned membrane surface and the one without. For applied potentials on the order of a few thermal volts or less, the patterned system has a lower current density than the unpatterned one due to the smaller permeable surface area available. As Δ*V* is increased, no initial limiting plateau due to the diffusion limit occurs as in the unpatterned system because the patterning induces vortices in a thresholdless manner as described previously^27^. At low voltages, one vortex pair per pattern period is generated (Fig. 6(a)). These vortices are driven at the intersections between permeable and impermeable regions with strong outflow over the membrane and inflow over the impermeable zone. They provide additional advective transport, even at voltages lower than the critical voltage, thereby preventing a plateau at the traditional limiting current. Instead, a new type of plateau occurs between 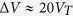 and 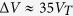 due to changes in the vortex structure.

As can be seen in Fig. 6(b) and Supplementary Movie S5, at Δ*V* = 20*V*_*T*_ a second pair of small counter-rotating vortices develops on each membrane region. These secondary vortices grow in size with increasing applied potential (Fig. 6(c)) while the initial vortices shrink. This shrinkage of the large scales reduces the effectiveness of the system for transporting current thus causing the novel secondary plateau in the IV curve. At Δ*V* = 35*V*_*T*_, the primary and secondary vortices are very close in size and generate similar currents, within ≈20%. This is in contrast to an approximately factor of 3 difference at Δ*V* = 20*V*_*T*_. A jump by a factor of almost two in the current between Δ*V* = 35*V*_*T*_ and Δ*V* = 40*V*_*T*_ is due to the onset of instability at a new, shifted, 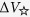. For Δ*V* = 40*V*_*T*_ (Fig. 6(d) and Supplementary Movie S6), after an initial transient, the system reaches a statistically stationary state dominated by the small-scale convective instability on the membrane surfaces and one large counter-rotating vortex pair induced per pattern period. The large scales are subject to a low frequency overturning motion which causes large temporal current fluctuations. Δ*V* = 50*V*_*T*_, Δ*V* = 60*V*_*T*_ (Fig. 6(e) and Supplementary Movie S7), and Δ*V* = 70*V*_*T*_ exhibit qualitatively similar behavior, however, at these voltages the large scales do not overturn. The regularization of the large-scale flows is responsible for the dramatic amplification of the current density relative to the homogeneous system in this voltage range (Fig. 5).

For higher applied voltages Δ*V* = 80*V*_*T*_ (Supplementary Movie S8) and 100*V*_*T*_ (Fig. 6(f)), the EKI driven chaos begins to dominate the pattern-induced modes, and thus the patterned and unpatterned systems behave in a similar manner. The effect of this transition from strongly interacting to chaos dominated on the IV curve is the peak at about Δ*V* = 70*V*_*T*_ as the reduction in current caused by the reduced effectiveness of the unregulated vortices in transport is balanced by the increasing flow and electromigration velocities driven by higher potentials. At large enough applied potentials, the reduction in active surface area will lead to lower current densities for the patterned system with a crossover point near Δ*V* = 100*V*_*T*_ seen in Fig. 5. Representative surface-averaged current versus time curves for various voltages are in Supplementary Fig. S1.

### Pattern Optimization

A second type of pattern optimization investigated, in addition to pattern size, is the effect of different fractions of the pattern consisting of membrane 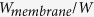 and impermeable 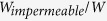 materials. Variation of the current density with the permeable fraction of the lower surface at Δ*V* = 20*V*_*T*_ is shown in Fig. 7 for a fixed pattern period. Perhaps unexpectedly, the peak current is seen for an asymmetric pattern with a smaller permeable region (40%) than impermeable (60%) with a relative increase of approximately 20% in the current density over the symmetric pattern and roughly 80% higher than that of the homogeneous membrane. The optimal pattern ratio is most likely voltage and pattern size dependent. We leave further investigation of this combined optimization for future studies.

## Discussion

We have shown that patterning of ion-selective surfaces with impermeable regions has the potential to enhance transport to ion-selective surfaces by a factor of almost two. In the underlimiting regime, we have found that mixing due to second-kind electroosmosis induced by the patterns eliminates the traditional diffusion-limited current, and instead a new limiting regime due to changes in vortex structure occurs. For larger voltages, when the system is unstable, the pattern-induced flows regularize the flows generated by EKI and enhance net transport. Also, our results indicate that pattern width and aspect-ratio optimization is a promising avenue for improving ion-selective surfaces.

The presented results have important implications for the understanding and design of ion-selective surfaces. They provide insights into the mechanisms responsible for the enhancements in current seen in studies of inhomogeneous and patterned membranes. They also indicate that patterns should be designed to scale with the diffusion layer size. Although we have focused on the ability of patterning to enhance transport, we have also shown that patterning could be used to suppress EKI in systems where it is unwanted. Additionally, the coexistence regime found for homogeneous membranes provides another possible source for the uncertainty seen in experimental studies, however further study is needed to see whether this regime also exists in 3D. Other significant future areas of research include three-dimensional effects both for optimized 1D and 2D surface patterns and modeling nonidealities in both realistic membranes and surface patterns.

## Methods

We here briefly discuss the numerical methods used in our simulations. The equations are discretized in space using second-order accurate finite differences on a staggered, structured mesh, and in time using a second order backward difference formula. In order to resolve the thin EDLs near the membranes, a hyperbolic tangent function is used to properly stretch the mesh in the wall-normal direction. Efficient solution of the species transport equations is done using an iterative semi-implicit method where only the wall-normal transport terms are updated implicitly while the tangential terms are solved explicitly. By treating these terms implicitly, we stabilize the small numerical diffusion time-scale within the EDLs while still using fine enough timesteps to resolve all dynamically relevant scales. Further details of this method can be found in Karatay *et al*.^19^. The only minor difference between our method and that of^19^ is that in our implicit treatment the Poisson equation is directly included coupled with the electromigration fluxes, while in^19^ the effect of the Poisson equation is analytically embedded in the discretization of the electromigration fluxes. Our approach, which requires solving a slightly larger band-width matrix, uncouples the numerical conservation criteria for the ionic species from the solver convergence criteria. We found this approach to be critical for our system, since certain species must be globally conserved, thus even slight leakages due to numerical errors will, over time, lead to nonphysical results. The Poisson and Poisson-like equations for *ϕ* and ***u*** are solved using Fourier transforms in the periodic tangential direction. A parallel numerical code was written and verification performed via the method of manufactured solutions^30^.

**Figure 1:**
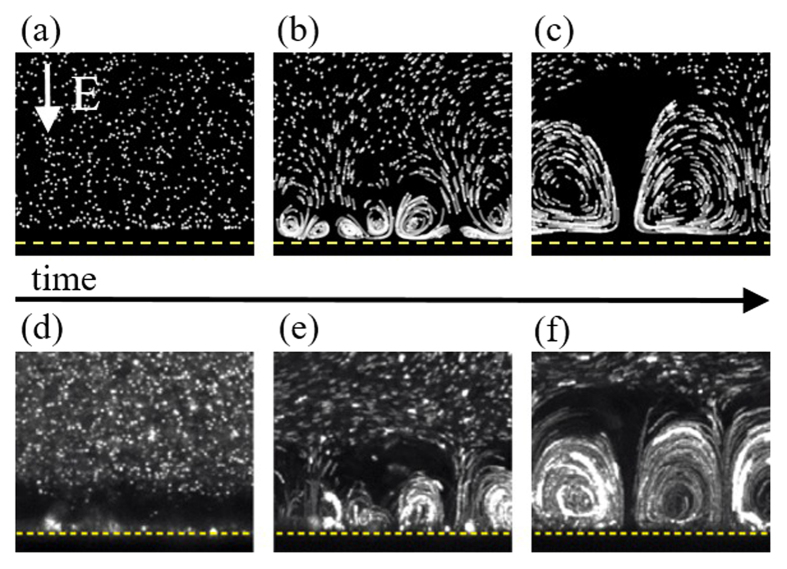
Snapshots of simulated (**a**–**c**) and experimental (**d**–**f**) tracer particle fields near a homogeneous cation selective membrane. In all images the dashed lines indicate the membrane surface. Simulations were performed at a constant voltage of 40*V*_*T*_, experiments at a current density of 10 A/m^2^. Simulated and experimental particles have a zeta potential of −1 mV. Images (**d**–**f**) adapted with permission from de Valença *et al*.^2^ Copyrighted by the American Physical Society.

**Figure 2:**
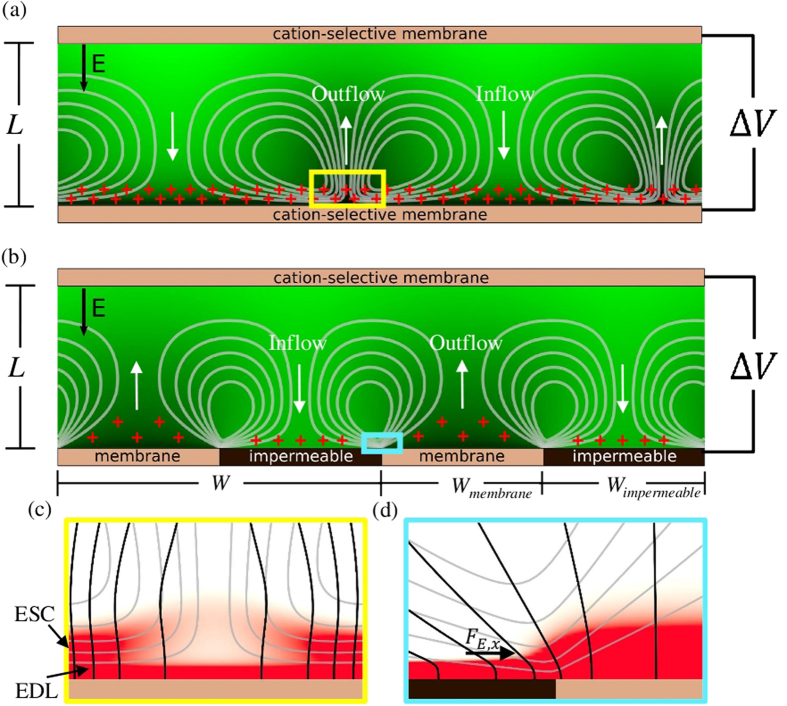
Schematic showing the two model problems investigated. (**a**) Upper and lower surfaces are both cation-selective membranes. (**b**) The lower surfaces is patterned with membrane (ion-permeable) and impermeable regions. In both (**a**,**b**), the color field represents species concentration with brighter (green) colors indicating higher concentrations. Cation and anion concentration fields are visually identical when plotted at this scale due to local electroneutrality. (**c**,**d**) Show charge-density fields zoomed in on the boxes (yellow and blue respectively) in (**a**,**b**). Light gray lines are flow streamlines, and black lines are electric field lines.

**Figure 3:**
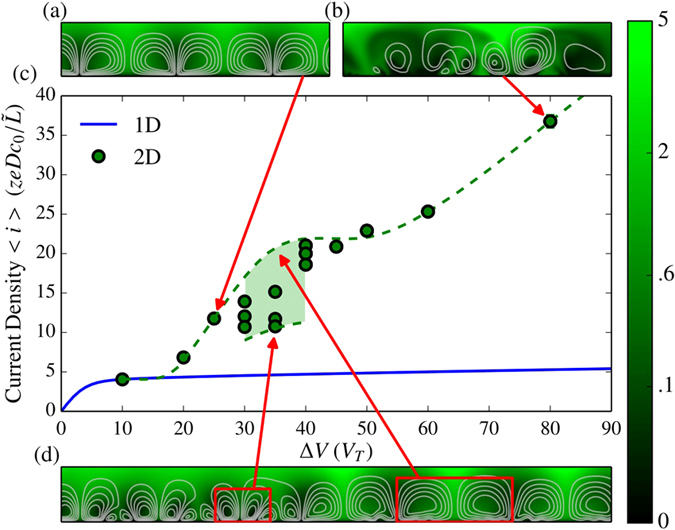
Effect of different modes of EKI on the time-averaged current density through a homogeneous membrane. (**a**,**b**,**d**) Instantaneous species concentration fields with flow streamlines. At Δ*V* = 25*V*_*T*_ and 80*V*_*T*_, (**a**,**b**) respectively, single dynamical modes are present. Boxed regions in (**d**) Δ*V* = 35*V*_*T*_ correspond to regions of small and large vortices representative of those from which the current bounds were extracted as depicted by the arrows. (**c**) Averaged current density vs applied voltage for 1D (solid blue line) and 2D (circles) simulations.

**Figure 4:**
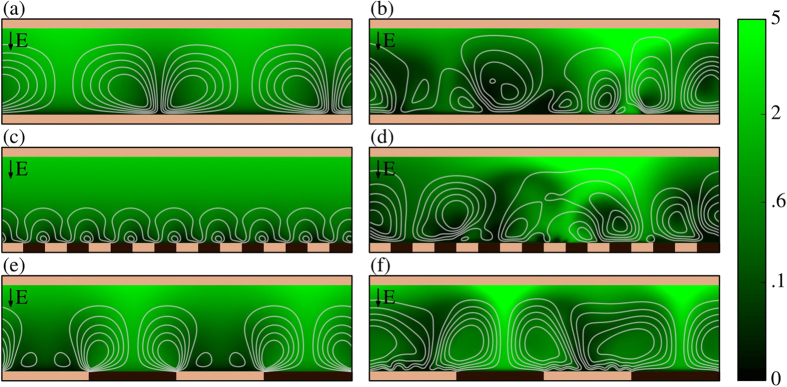
Qualitative effect of small and large pattern wavelengths on species concentrations. Instantaneous species concentration fields with flow streamlines plotted on top for three surface geometries at voltages of 20*V*_*T*_ (left column) and 60*V*_*T*_ (right column), and indicating the locations of impermeable (dark colored) and membrane (light colored) regions on the patterned surface.

**Figure 5:**
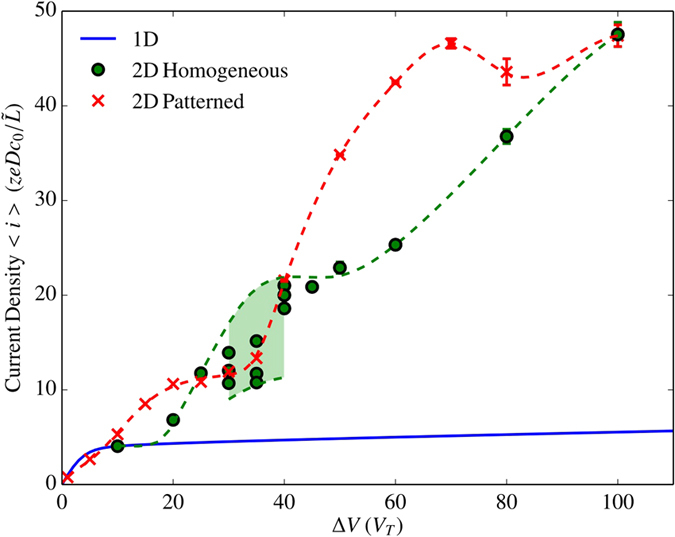
Effect of impermeable surface patterning on current versus voltage curves. IV curves for three different cases are shown. First is a one-dimensional model meaning that there is no flow. Second is a two-dimensional model with homogeneous membranes at both walls. Third is a two-dimensional model where the lower surface is patterned as shown in Fig. 2(b).

**Figure 6:**
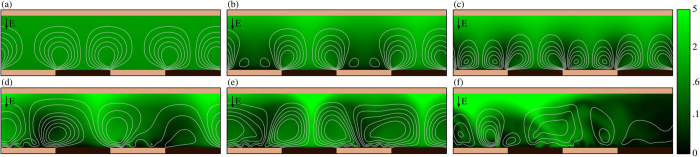
Effect of applied potential on systems with patterned surfaces. Shown are instantaneous species concentration fields with flow streamlines at (**a**) Δ*V* = 1, (**b**) Δ*V* = 20, (**c**) Δ*V* = 30, (**d**) Δ*V* = 40, (**e**) Δ*V* = 60, (**f**) Δ*V* = 100.

**Figure 7:**
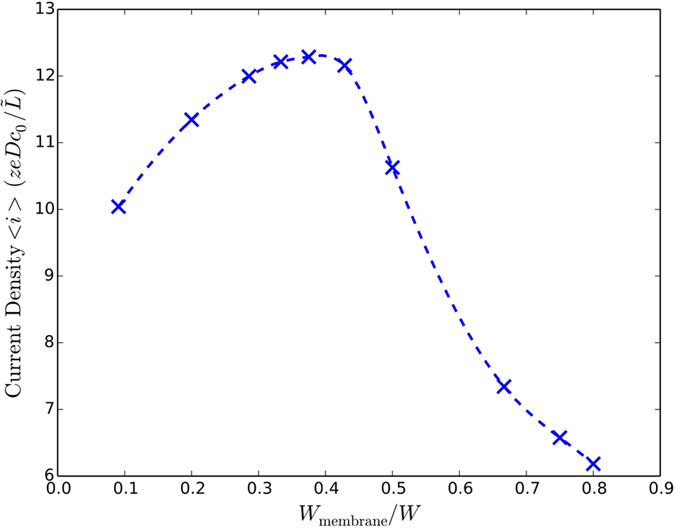
Optimization of pattern permeable fraction. Variation of steady-state current density with respect to membrane fraction for a fixed pattern length of *W* = 2*L* and applied voltage of 20*V*_*T*_.

The visualizations shown in Fig. 1(a–c) were generated using what we term virtual colloidal particles. In this method, which is performed as a post-processing step, particles are initalized randomly throughout the domain. Particles are then advected by the flow and undergo electrophoresis with a velocity determined according to the Helmholtz-Smoluchowski equation. Specifically, this means solving in dimensionless units 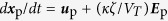 where *ζ* is the particle zeta potential, ***x***_p_ is the position of the p^th^ particle, and ***u***_p_ and ***E***_p_ are the local flow velocity and electric field respectively. Particle paths are then visualized over a time interval Δ*t* so that the length of the tail shown behind each particle is proportional to its velocity.

## Additional Information

**How to cite this article**: Davidson, S. M. *et al*. On the Dynamical Regimes of Pattern-Accelerated Electroconvection. *Sci. Rep.* **6**, 22505; doi: 10.1038/srep22505 (2016).

## Supplementary Material

Supplementary Information

Supplementary Movie S1

Supplementary Movie S2

Supplementary Movie S3

Supplementary Movie S4

Supplementary Movie S5

Supplementary Movie S6

Supplementary Movie S7

Supplementary Movie S8
