# Supplementary material for: On the Dynamical Regimes of Pattern-Accelerated Electroconvection
Source: Sci Rep. 2016 Mar 3;6:22505. doi: 10.1038/srep22505 (PMC4776150; doi:10.1038/srep22505)
Supplement: Supplementary Information [file srep22505-s1.pdf]

t

Supplementary Information:

On the Dynamical Regimes of Pattern-Accelerated Electroconvection

Scott M Davidson[1], Matthias Wessling[2,3], and Ali Mani[1]

This document contains additional useful information obtained from post-processing of our DNS data. These include, current versus time data for both patterned and homogeneous membranes as well as quantitative analysis of vortex size distribution and associated current densities for homogeneous membranes. Additionally, movies from our simulations demonstrating evolution of flow path-lines and ion concentration fields are provided in the accompanying movie files.

Supplementary Figure S1 illustrates surface-averaged current versus time data for a patterned membrane with pattern period of $w = 2L$. As shown for voltages up to 30 thermal volts the signal reaches a steady value after an initial transience. Correspondingly steady vortices are formed in these cases. For higher voltages the steady solution is unstable and a chaotic current signal is observed.

Supplementary Figure S2 shows the same measurement for a homogeneous membrane. Here up to voltage of ~ 20 thermal volts the system is stable without any vortices (1D), and steady vortices are sustained above this threshold and up to voltage of ~ 40. Beyond this voltage chaotic flow and current signals are observed.

For homogeneous membranes, and in the voltage range of ~ 30 to ~ 40 thermal volts, multiple vortex sizes are generated as the system response, which can often coexist. This coexistence leads to a range of possible steady current densities depending on the fraction of the domain occupied by each vortex size. We determined the approximate upper and lower current density limits for large and small vortices by picking out regions of large and small vortices and then averaging current densities over these regions. This process was performed by first measuring the charge density versus x at a selected y location as seen in Supplementary Figure 3. Then, vortex pair centers were found by looking for peaks in the charge density above a threshold value. The fact that these peaks correspond to vortex pair centers can be seen visually in the bottom part of Supplementary Figure 3. Once vortex pair centers were found, then distances between adjacent centers could be found. These distances exhibit a clear bimodal distribution as shown in Supplementary Figure 4. Based on this distribution, each vortex pair, and hence region of surface was classified as either “large” or “small”. Once the surface was separated into these two classes, conditional means of the surface current density for each class were taken to find the current density of each class, i.e. the large and small vortex current density. By performing this procedure for multiple snapshots in time and averaging over both time and various simulation replicas, approximate statistics, including upper and lower bounds for current, were found.

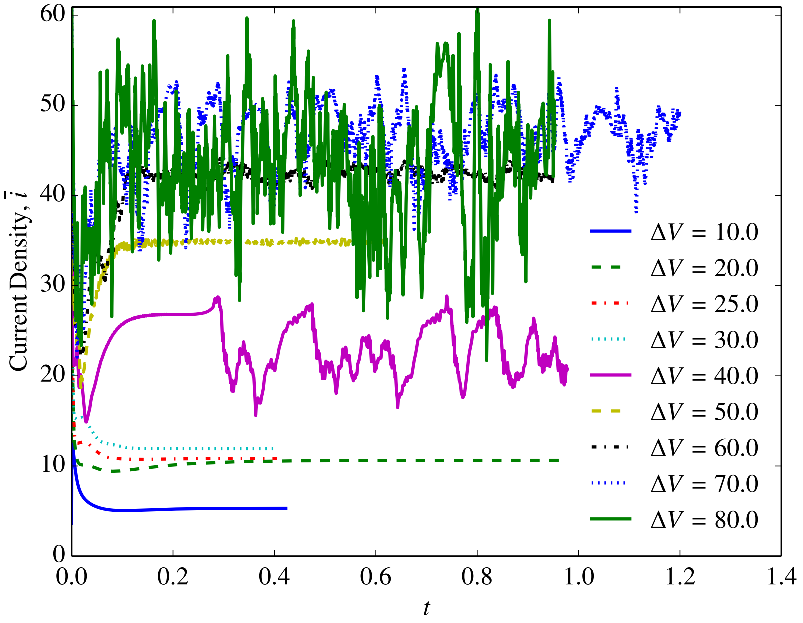

Supplementary Figure 1: surface-averaged current versus time for a patterned membrane surface at different applied voltages

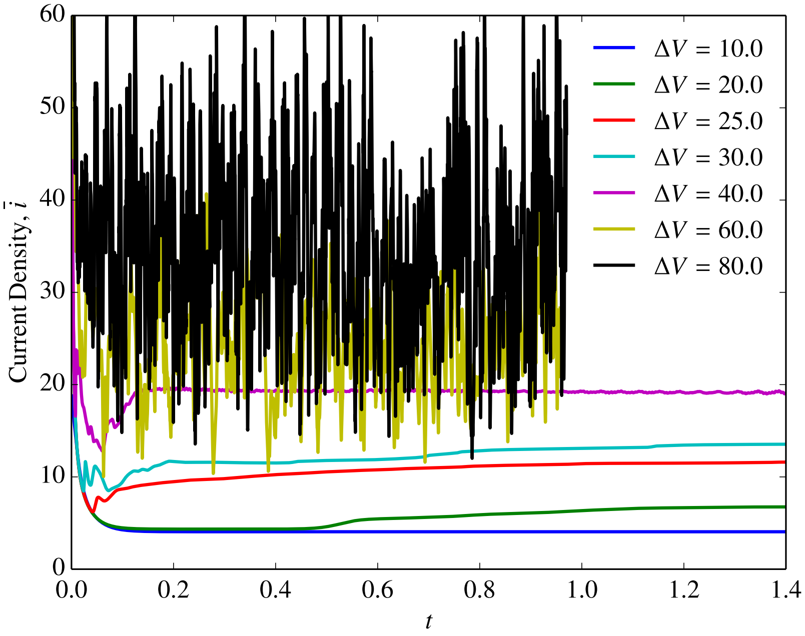

Supplementary Figure 2: surface-averaged current versus time for a homogeneous membrane surface at different applied voltages.

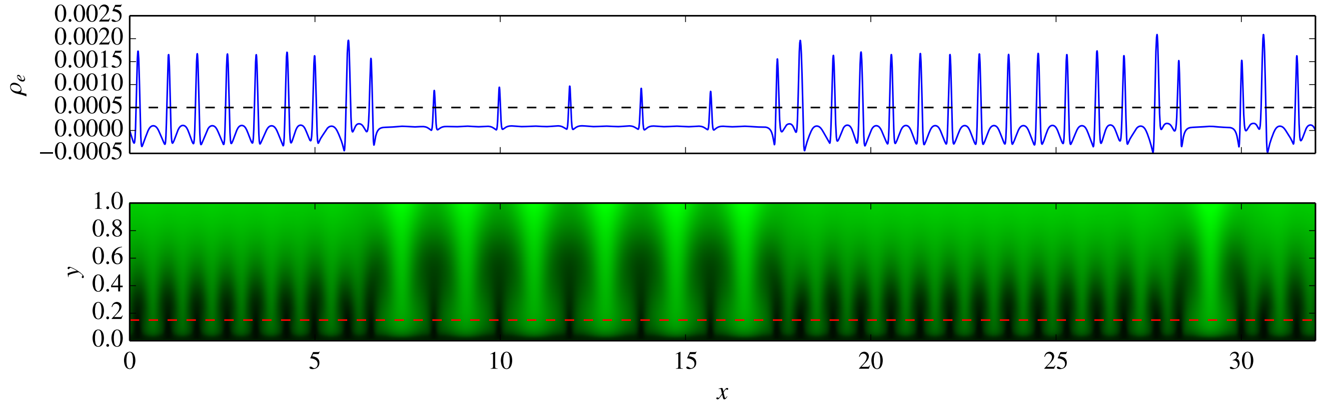

Supplementary Figure 3: top: charge density versus x at $y = 0.15$ corresponding to the dashed red line in the bottom plot. The black line indicates the threshold charge density used for finding peaks. Bottom: cation concentration field at the same time. Note that the bottom figure is compressed in the x −direction.

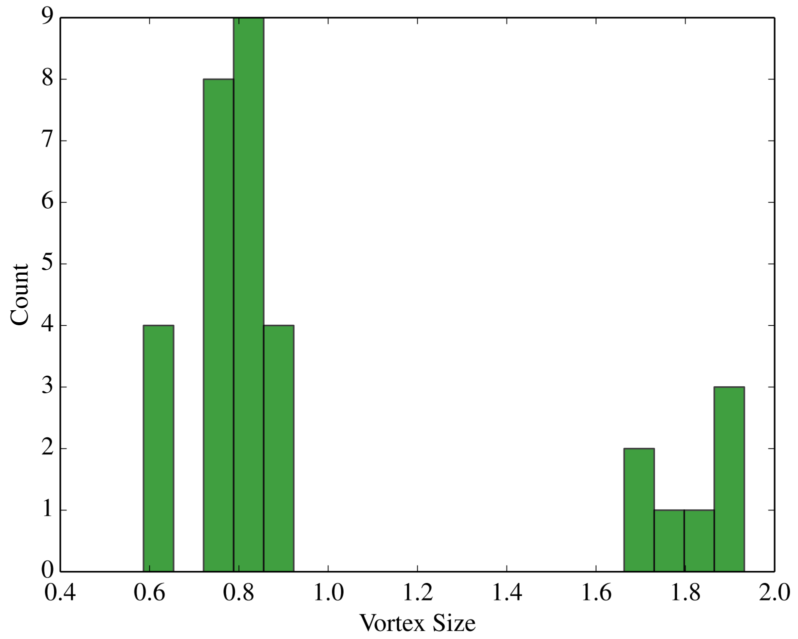

Supplementary Figure 4: histogram of vortex sizes

List of legend for Supplementary Videos

Supplementary Movie S1: Flow and concentration fields near a homogeneous membrane for an applied potential of 20 thermal volts. Movie shows the cation concentration in green and fluid flow is visualized with numerical tracer particles. Time is presented in units of L^2/D.

Supplementary Movie S2: Flow and concentration fields near a homogeneous membrane for an applied potential of 40 thermal volts. Movie shows the cation concentration in green and

fluid flow is visualized with numerical tracer particles. Time is presented in units of L^2/D.

Supplementary Movie S3: Flow and concentration fields near a homogeneous membrane for an applied potential of 60 thermal volts. Movie shows the cation concentration in green and fluid flow is visualized with numerical tracer particles. Time is presented in units of L^2/D.

Supplementary Movie S4: Flow and concentration fields near a homogeneous membrane for an applied potential of 80 thermal volts. Movie shows the cation concentration in green and fluid flow is visualized with numerical tracer particles. Time is presented in units of L^2/D.

Supplementary Movie S5: Flow and concentration fields near a patterned membrane for an applied potential of 20 thermal volts. Movie shows the cation concentration in green and fluid flow is visualized with numerical tracer particles. Time is presented in units of L^2/D.

Supplementary Movie S6: Flow and concentration fields near a patterned membrane for an applied potential of 40 thermal volts. Movie shows the cation concentration in green and fluid flow is visualized with numerical tracer particles. Time is presented in units of L^2/D.

Supplementary Movie S7: Flow and concentration fields near a patterned membrane for an applied potential of 60 thermal volts. Movie shows the cation concentration in green and fluid flow is visualized with numerical tracer particles. Time is presented in units of L^2/D.

Supplementary Movie S8: Flow and concentration fields near a patterned membrane for an applied potential of 80 thermal volts. Movie shows the cation concentration in green and fluid flow is visualized with numerical tracer particles. Time is presented in units of L^2/D.
